# Venlafaxine-induced hypoglycemia

**DOI:** 10.1503/cmaj.78409

**Published:** 2021-04-19

**Authors:** Jane Kobylianskii, Peter E. Wu

**Affiliations:** Internal medicine resident, Department of Medicine, University of Toronto, Toronto, Ont.; Internal medicine and clinical pharmacology/toxicology specialist, Division of Clinical Pharmacology & Toxicology, Department of Medicine, University of Toronto, Toronto, Ont.

Murphy and colleagues present a valuable case, highlighting the severe toxicity from the overdose of a common antidepressant. 1 This case also draws attention to the underappreciated adverse effect of venlafaxine-induced hypoglycemia.

Their patient developed severe hypoglycemia requiring intravenous dextrose. Though the hypoglycemia was attributed to acute hepatic injury,^1^ it is very possible that this was, in fact, secondary to the venlafaxine overdose itself. Several case reports describing venlafaxine-induced hypoglycemia have been published.^2^–^4^ Although the exact mechanism is yet to be elucidated, it is postulated that hypoglycemia develops through μ-opioid receptor–mediated processes that reduce hepatic gluconeogenesis and increase peripheral glucose uptake and insulin sensitivity.^4^

This rare adverse event is supported by the molecular similarity of venlafaxine to the analgesic tramadol, a more established and recognized cause of hypoglycemia. 4,5 Animal models show that the administration of tramadol causes plasma glucose levels to drop because of enhanced peripheral glucose uptake and glycogen synthesis, reduced hepatic gluconeogenesis and increased hepatic insulin sensitivity.^5^ These effects are thought to be a μ-opioid receptor–mediated process.^5^ Venlafaxine and tramadol are nearly identical in structure and share chemical properties, supporting a similar mechanism of causing hypoglycemia.^4^

Hypoglycemia is a rare, underappreciated adverse effect of venlafaxine that should be considered and treated appropriately, particularly in overdose situations.
